# Correction: Evaluating the effectiveness of integrating biofeedback in the treatment of aggressive outbursts (BRET-IA2): A study protocol

**DOI:** 10.1371/journal.pone.0336635

**Published:** 2025-11-10

**Authors:** Alberto J. Molina-Cantero, Isabel Rojas-Pérez, Montserrat Gómez de Terreros-Guardiola, Isabel Gómez-González, José C. Vidosa-Batllés, Teresa de Jesús Bermejo-González, Manuel Merino-Monge

The Funding statement for this article is incorrect. The correct Funding statement is as follows: This publication is part of the project PID2023–147508OB-I00, funded by MICIU/AEI/ 10.13039/501100011033 and by ERDF/EU awarded to A.J.M.C & M.M.M. (BRETIA2 project). The funders had no role in study design, data collection and analysis, decision to publish, or preparation of the manuscript.
