# Supplementary material for: Evaluating the effectiveness of integrating biofeedback in the treatment of aggressive outbursts (BRET-IA2): A study protocol
Source: PLoS One. 2025 Jul 7;20(7):e0327361. doi: 10.1371/journal.pone.0327361 (PMC12233311; doi:10.1371/journal.pone.0327361)
Supplement: S1 File — (PDF) [file pone.0327361.s002.pdf]

UNIVERSIDAD DE SEVILLA 1505

VICE-RECTORATE FOR RESEARCH

Research Ethics Committee

OPINION OF THE UNIVERSITY OF SEVILLE RESEARCH ETHICS COMMITTEE

Ms. Fátima Chacón Borrego, Secretary of the

University of Seville Research Ethics Committee

CERTIFIES

That this Committee has evaluated the proposal of Mr. Alberto Jesús Molina Cantero, as principal investigator of the research project entitled ***"The use of Biofeedback and virtual reality as tools for the treatment of aggressive responses in adolescents"*** with internal code (PEIBA) of protocol: 1075-M1-24.

- Protocol: version M1 dated 10/05/2024
- HIP: M2 version dated 10/07/2023
- CI: M2 version dated 10/07/2024

AND CONSIDERS THAT:

The necessary requirements for the suitability of the protocol in relation to the objectives of the study are met and it complies with the ethical principles applicable to this type of studies and included in the Declaration of Helsinki.

The processing of participants' personal data is in accordance with the provisions of Regulation (EU) 2016/679 of the European Parliament and of the Council of 27 April 2016 and Organic Law 3/2018 of 5 December on the Protection of Personal Data and guarantee of digital rights.

The procedure for obtaining informed consent is adequate. In view of the

above:

The *Research Committee of Seville,* at its meeting held on June 26, 2024
(Act 08/24) after evaluation of the aforementioned study issues a **FAVORABLE OPINION.**
This Committee is constituted and acts in accordance with the regulations in force and the guidelines of the International Conference on Good Clinical Practice.

Secure Verification Code	bvCR4m4FEnT1i1dRYlpJig== bvCR4m4FEnT1i1dRYlpJig==	Date	24/07/2024
Signed By	FATIMA CHACON BORREGO		
Verification Url	https://pfirma.us.es/verifirma/code/bvCR4m4FEnT1i1dRYlpJig%3D%3D	Page	1/2

ANNEX

COMMITMENTS MADE BY THE RESEARCHER WITH RESPECT TO THE RESEARCH ETHICS COMMITTEE OF THE UNIVERSITY OF SEVILLE

The principal investigator is reminded that the execution of the research project entails the following commitments:

1. Execute the project in accordance with what is specified in the protocol, both in scientific and ethical aspects.

2. Notify the Committee of all modifications or amendments to the project and request a re-evaluation of relevant amendments (e.g., modifications affecting objectives, sample, data collection and processing strategies, etc.).

3. Send an abbreviated final report to the Committee at the end of the project. This report should include the following sections following the model proposed by the CEIUS available at https://investigacion.us.es/investigacion/comites-eticos/ceius:

- Project registration number in CEIUS and in public databases of research projects, if applicable.
- Brief summary of the impact of the project (scientific, social and technological/economic), including the main findings and/or conclusions, as well as the type and mode of information transmitted to the project subjects about the results that directly affect their health.
- List of scientific publications generated by the project.

4. Initiate the procedure for the identification, approval, and publication of the data processing in question for which you should contact the Data Protection Officer of the University of Seville (dpd@us.es).

The Committee, in the exercise of its functions, may carry out random monitoring of projects during their execution or at the end of the project.

Secure Verification Code	bvCR4m4FEnT1i1dRYlpJig== bvCR4m4FEnT1i1dRYlpJig==	Date	24/07/2024
Signed By	FATIMA CHACON BORREGO		
Verification Url	https://pfirma.us.es/verifirma/code/bvCR4m4FEnT1i1dRYlpJig%3D%3D	Page	2/2
