# Supplementary material for: Evaluating the effectiveness of integrating biofeedback in the treatment of aggressive outbursts (BRET-IA2): A study protocol
Source: PLoS One. 2025 Jul 7;20(7):e0327361. doi: 10.1371/journal.pone.0327361 (PMC12233311; doi:10.1371/journal.pone.0327361)
Supplement: S2 File — (PDF) [file pone.0327361.s003.pdf]

VICE RECTORATE FOR RESEARCH

Directorate of the Research Secretariat

Research Ethics Committee of the

University of Seville

REQUEST FOR A REPORT ON ETHICAL ISSUES TO THE US RESEARCH ETHICS COMMITTEE

Internal Code:	Version:	Date of entry:

I.- RESEARCHER'S DATA

Principal Investigator:	
Name and Surname	*ALBERTO JESÚS MOLINA CANTERO*
Professional Category	*TENURED UNIVERSITY LECTURER*
Work Center:	*COMPUTER DE SEVILLE- ETS ENGINEERING UNIVERSITY*
Qualification:	*COMPUTER ENGINEERING*

Researcher 1:	
Name and Surname	*ISABEL ROJAS PÉREZ*
Professional Category	*PSYCHOLOGIST*
Work Center:	*INEBIR*
Qualification:	

Researcher 2:	
Name and Surname	*MANUEL MERINO MONGE*
Professional Category	*PHYSICIAN ASSISTANT*
Work Center:	*COMPUTER DE SEVILLE- ETS ENGINEERING UNIVERSITY*
Qualification:	*COMPUTER ENGINEERING*

Researcher 3:	
Name and Surname	*ISABEL MARÍA GÓMEZ GONZÁLEZ*
Professional Category	*TENURED UNIVERSITY LECTURER*
Work Center:	*COMPUTER DE SEVILLE- ETS ENGINEERING UNIVERSITY*
Qualification:	*COMPUTER ENGINEERING*

Researcher 4:	
Name and Surname	*JUAN ANTONIO CASTRO GARCÍA*
Professional Category	*TEMPORARY SUBSTITUTE TEACHER*
Work Center:	*COMPUTER DE SEVILLE- ETS ENGINEERING UNIVERSITY*
Qualification:	*COMPUTER ENGINEERING*

II.- DATA IDENTIFYING THE RESEARCH PROJECT

Title	THE USE OF BIOFEEDBACK, VIRTUAL REALITY AND COGNITIVE RESTRUCTURING IN THE TREATMENT OF AGGRESSIVE RESPONSES IN CHILDREN AND ADOLESCENTS						
Type	X	P. Research		T.D.		T.F.M.		T.F.G.
Financing	*NO*						
Submitted to a public call for proposals:							
Center where the project will be carried out	*INEBIR*						

III. RESEARCH PROTOCOL

III.1. JUSTIFICATION	(Expose the importance y topicality of importance and topicality of the subject. Main practical contributions practices that justify the realization of the project. Bibliography relevant recent)

Several disorders affecting behavior, such as ASD, intermittent explosive disorder (IED) or attention deficit hyperactivity disorder (ADHD), are characterized by a reduction in the ability to control/process emotions and behaviors that can end in aggressive episodes [1]-[2]. Our interest is mainly focused on providing the individual with the skills and tools to identify his or her internal state, anticipate and avoid the aggressive outburst.

The emotions experienced when interacting with the environment involve certain physiological arousal, which is manifested by activation/deactivation of the sympathetic nervous system (SNS) and parasympathetic nervous system (PNS). Consequently, some organs, innervated by these branches, show an increase/decrease in their activity, which could help those who have difficulty in identifying emotions to do so. For example, the heart rate is accelerated/decreased depending on the level of activity of the SNS and PNS [3].

The use of physiological signals, which are continuously displayed to patients, as part of a feedback process, can be very beneficial. Biofeedback is a well-known technique that has been tested in several areas, such as ADHD, to improve children's attention [4-5]. Along with the use of biofeedback, virtual reality (VR) and specifically, immersive virtual reality (IVR), has demonstrated its efficacy in therapy in behavioral disorders, improving subjects' cognitive skills, attention and improving emotion identification [6-8]. IVR allows to reproduce, in a safe environment, everyday life situations in which they can practice and develop social skills while eliminating the anxiety that the real environment can provoke.

Cognitive restructuring therapies [9-11] have been found to be effective for anger management. They are based on the fact that people minimize their negative feelings and behaviors when they are aware of how irrational they are and work to change their mind by continuously confronting them. Virtual reality and biofeedback are tools that will help the patient to have a better way of recognizing their emotions, which reinforces the cognitive restructuring therapy. Specifically, what is proposed is that, through cognitive restructuring, we can modify the way we think and interpret things. Many times, a good part of the problems are related to the impossibility of looking for alternative explanations about what is happening. Thus, cognitive restructuring can be interpreted as a strategy to improve the chances of patients modifying their cognitive schemas in the most adaptive way possible. In other , cognitive restructuring therapy is focused on the subject's ability to identify the thoughts and

negative beliefs presented in situations of conflict and/or frustration, which are the main triggers of explosive behaviors. They will be taught how certain physiological responses have been conditioned to certain cognitions that cause the person to lose self-control. Therefore, they are trained in the identification of physiological response, the identification of the emotion that triggers and the negative thinking that accompanies them.

[1] M. Schonenberg, A. Schneidt, E. Wiedemann, and A. Jusyte, "Processing of dynamic affective information in adults with adhd," Journal of attention disorders, vol. 23, no. 1, pp. 32-39, 2019.

[2] M. K. Fahlgren, A. A. A. Puhalla, K. M. Sorgi, and M. S. McCloskey, "Emotion processing in intermittent explosive disorder," Psychiatry research, vol. 273, pp. 544-550, 2019.

[3] B. M. Appelhans and L. J. Luecken, "Heart rate variability as an index of regulated emotional responding," Review of general psychology, vol. 10, no. 3, pp. 229-240, 2006.

[4]Y.-C. Liao, N.-W. Guo, B.-Y. Su, S.-J. Chen, H.-F. Tsai, and K.-Y. Lee, "Frontal beta activity in the meta-intention of children with attention deficit hyperactivity disorder," Clinical EEG and Neuroscience, vol. 52, no. 2, pp. 136-143, 2021.

[5] E. Shereena, R. Gupta, C. Bennett, K. Sagar, and J. Rajeswaran, "Eeg neurofeedback training in children with attention deficit/hyperactivity disorder: a cognitive and behavioral outcome study,"

Clinical EEG and neuroscience, vol. 50, no. 4, pp. 242-255, 2019.

[6]V. Delvigne, L. Ris, T. Dutoit, H. Wannous, and J.-P. Vandeborre, "Vera: Virtual environments recording attention," in 2020 IEEE 8th International Conference on Serious Games and Applications for Health (SeGAH). IEEE, 2020, pp. 1-7.

[7]S. Adabla, L. Nabors, and K. Hamblin, "A scoping review of virtual real-ity interventions for youth with attention-deficit/hyperactivity disorder," Advances in Neurodevelopmental Disorders, vol. 5, no. 3,

pp. 304-315, 2021.

[8] B. Karami, R. Koushki, F. Arabgol, M. Rahmani, and A.-H. Vahabie, "Effectiveness of virtual/augmented reality-based therapeutic interventions on individuals with autism spectrum disorder: A comprehensive meta-analysis," Frontiers in Psychiatry, vol. 12, p. 665326, 2021.

[9] L. Canet-Juric, A. Garc, M. L. Andr, S. Vernucci, Y. Aydmune, F. Stelzer et al., "Intervention on cognitive, behavioral and emotional self-regulation in children: A review of process-based and school curriculum approaches in Argentina," Revista Argentina de Ciencias del Comportamiento, vol. 12, no. 1, pp. 1-25, 2020.

[10] Bados, Arturo, and E. Garcia. "The cognitive restructuring technique." *Department of Personality, Psychological Assessment and Treatment. Faculty of Psychology, University of Barcelona* 15 (2010).

[11] Froján Parga, María Xesús, and Ana Calero Elvira. "Guide for the use of cognitive restructuring as a shaping procedure." *Behavioral Psychology* (2011).

III.2. METHODOLOGICAL ASPECTS

Objectives and Hypotheses (if applicable)

The main objective of this study is:

To verify the efficacy of the use of biofeedback and VR in the treatment of aggressive episodes with cognitive restructuring therapies.

As secondary objectives we propose the following:

2.a Analyze how an aggressive episode affects or influences physiological signals.
2.b Propose methods that allow predicting or anticipating the arrival of the

of an aggressive episode

Design:

Different sessions are proposed:

1. *S1. Information to parents and collection of informed consent*
2. *S2. In S1, parents are given a small camera hide in the main room. Up to four different episodes will be recorded. This session will be of varying length, depending on the frequency of aggressive episodes. Parents will edit the video to show only the previous minutes and the episode itself. Parents will be instructed on how to edit the video. Only the parents, the patient and psychologist of the research group will have access to the content of the recording.*
3. *S3. In this session, the parents and the therapist meet to recall the procedure to be followed for the following sessions. Strategies are chosen to facilitate the evocation of the conditions that led to the episode at home.*
4. *S4. This session has some initial actions that are shared with the following sessions. It consists of a baseline recording of the physiological signals when entering the clinic, a relaxation phase using virtual reality, and then a phase in which the conditions for generating an aggressive episode in the clinic are reproduced. For this, one of the videos will be used at random. In this session all possible physiological signals are measured, which are described in detail in the materials section. This is followed by a relaxation phase until the end of the session.*

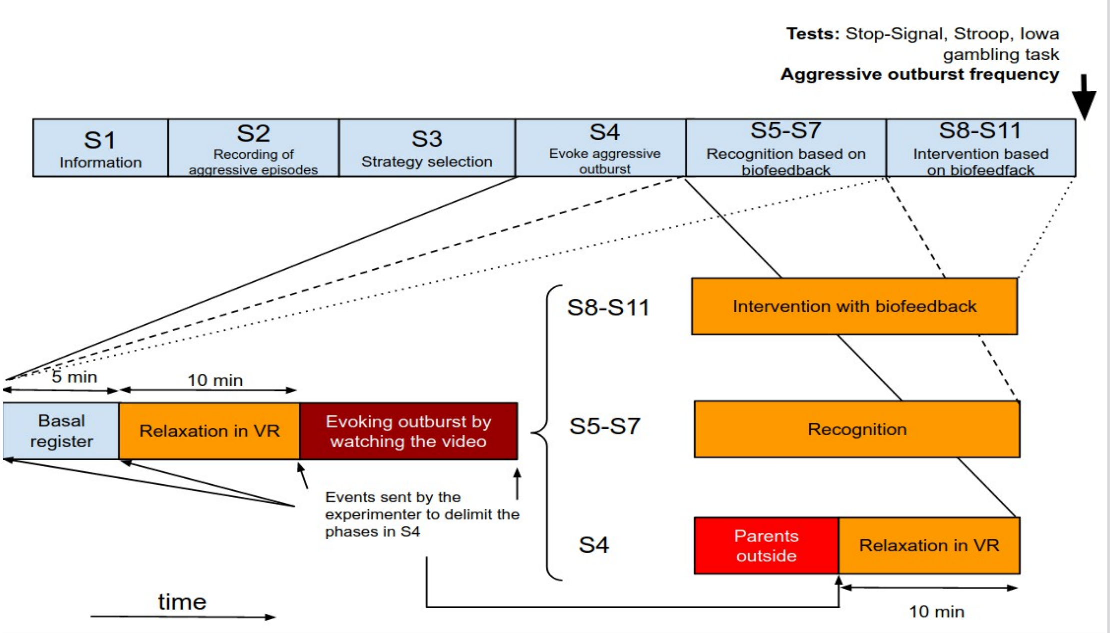

5. Sessions S5-S7 are structured identically to session S4 at the beginning, except that only EDA and HR signals will be used biofeedback. The remainder of the session is based on recognizing physiological markers associated with the impulsive act.
6. Sessions S8-S11 follow the same philosophy as the previous sessions, but at the end the intervention or cognitive restructuring therapy based on biofeedback is applied. The intervention is explained below.

Participants:

	a.- Procedure for the selection of participants indicating their origin *(Include aspects to confidentiality and anonymity).*
	Participants will be recruited from two centers that treat impulsivity and aggressive events in the metropolitan area of Seville (Inebir clinic and Instituto Hispalense de Pediatría). Participants will be assigned to the control and intervention groups following a simple randomized procedure whereby a list of numbers without repetition (as many as participants) is generated with a random generator and pairs are assigned to the control group and odd numbers to the intervention group. The difference between the control and intervention groups is that the control group will not attend sessions S5-S7, and will not receive biofeedback or VR in sessions S8-S11.

	b.- Sample size	*(Calculation of the number of participants needed to be representative of the population studied as well as the number of dropouts or failures).*
	We estimate the participation of 60 children/adolescents. In order to have some reference of the demographics with respect to the matter at hand, we have	

found a study[1] showing that 7.8% of the adult population in the US has or has exhibited explosive behaviors related to lack of anger control. In another article this percentage drops to 2% when dealing with severe levels of anger in school-aged adolescents [2]. However, we prefer to follow This percentage, approximates us, or gives us a basis for estimating the sample size. Because there are not many references in this regard, we prefer to take an average of both percentages, and assume 5%. Using Cochrane's formula, it is known that for a confidence interval ranging from 10% to 5%, a sampling error of 5%, a study population between 52 and 73 individuals is needed. We have chosen 60 participants to find a balance between the goodness of the sample and the work required for the experimental process.

[1] Okuda M, Picazo J, Olfson M, Hasin DS, Liu SM, Bernardi S, Blanco C. Prevalence and correlates of anger in the community: results from a national survey. CNS Spectr. 2015 Apr;20(2):130-9. doi: 10.1017/S1092852914000182. PMID: 25831968; PMCID: PMC4384185.

[2] Govindan, Radhakrishnan & Manjula, Munivenkatappa & Anjanappa, Shamala.(2019). Prevalence and Expression of Anger among Adolescents. 10.13140/RG.2.2.31052.16008.

c.- Criteria for inclusion and exclusion of participants

Participants must meet the following inclusion criteria:

1. *Be between 10 and 16 years of age*
2. *Previous diagnosis of ADHD, Asperger's or oppositional defiant disorder.*
3. *Positive result in any of the following tests: CACIA [1], CAPI-A [2], Stroop [3] or WCST [4].*
4. *Have presented intermittent aggressive episodes (verbal or physical) with a frequency of at least once a week during the two months prior to the start of the experiment.*

Excluded will be those that report

1. *Pharmacological therapy in the previous month*
2. *Clinical history with bipolar or psychotic disorder*
3. *Cranioencephalic trauma with loss of consciousness greater than 60 minutes.*

Items 1 and 2 of the inclusion criteria, and items 1, 2 and 3 of the exclusion criteria, are obtained in a previous interview, where parents, in addition, report the frequency of aggressive episodes. Those candidates to receive the intervention will take the proposed tests (point 3 of the inclusion criteria).

[1] N. Soreni, J. Crosbie, A. Ickowicz, and R. Schachar, "Stop signal and conners' continuous performance tasks: test-retest reliability of two inhibition measures in adhd children," Journal of Attention Disorders, vol. 13, no. 2, pp. 137-143, 2009.

[2] E. A. Sjoberg and G. G. Cole, "Sex differences on the go/no-go test of inhibition," Archives of Sexual Behavior, vol. 47, no. 2, pp. 537-542, 2018.

[3] F. Scarpina and S. Tagini, "The stroop color and word test," Frontiers in psychology, vol. 8, p. 557, 2017.

[4] D. Brevers, A. Bechara, A. Cleeremans, and X. No, "Iowa gambling task (igt): twenty years after-.

	gambling disorder and igt," Frontiers in psychology, vol. 4, p. 665, 2013.

Materials to be used: *(if questionnaires or scales are used, indicate the authors and validation data. If equipment is used, indicate data on its reliability and validity).*
Scales and apparatus will be used. The scales have been described in the previous section and include CACIA [1], Stroop [3] in its Spanish adaptation [2] or Iowa gambling task [4]. They are passed at the beginning and end of the experiment. The scales will be passed starting the experiment and at the end of the experiment. The difference in the markers obtained will make it possible to verify the efficacy of the intervention.<br><br>*CACIA is a child and adolescent self-control questionnaire administered to children between the ages of 11 and 19 years. It self-control by means of four scales: Personal Feedback (PR), Reward Delay (RR), Criterial Self-Control (CCC), Processual Self-Control (PCC). It includes an additional scale of sincerity (S). The direct scores obtained are converted into percentiles (Pc), with the mean being Pc 50 and scores below Pc 25 being considered significant. STROOP is a test of colors and words that assesses the influence of interference as an indication of impaired inhibitory control. CAPI-A is a Questionnaire of Premeditated and Impulsive Aggressiveness in Adolescents that aims to study both premeditated and impulsive aggression. Finally, WCST or Wisconsin Card Sorting Test. K. Heaton et al. Spanish adaptation: V. de la Cruz López evaluates executive functions, such as planning, organized inquiries and use of environmental feedback to change schemes, with great sensitivity to assess the effects of frontal lobe alterations related to impulsivity. The Wisconsin test allows the assessment of executive functions, which can be altered in syndromes such as ADHD, which, in turn, can be a triggering factor for frequent aggressive behavior.*<br><br>*Regarding the equipment to be used, the graph below shows the set of materials to be used in the laboratory. The participant will normally wear type 2 and 3 wearable devices, and in one session, the OpenBCI.*

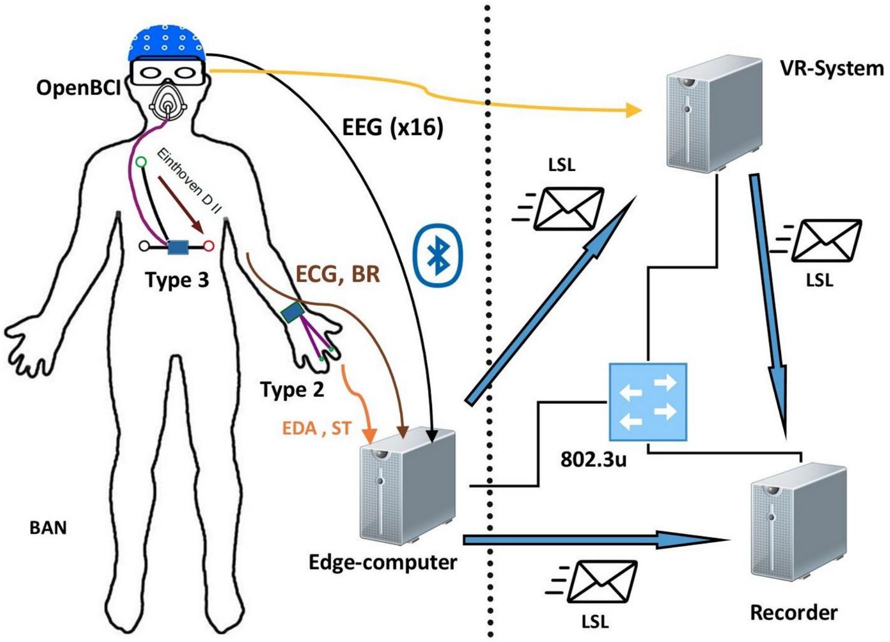

Type 2 and 3 wearable devices allow the measurement of different physiological variables such : electrocardiogram (ECG), respiratory rate (BR), skin conductance (EDA) and temperature (ST). These devices have been previously evaluated. The results can be seen in a recent publication [5]. For the measurement of EEG signals we will use OpenBCI, which is a low-cost solution based on a high quality ADS 1299ADC bioamplifier with 8 channels from Texas instruments. Its use has been scientifically validated[6] and appears in multiple papers in .

[1] Capafóns, A., and F. Silva. "Child and adolescent self-control questionnaire Rev.)." Madrid: TEA (2001)..

[2] Andreu, José Manuel. "Cuestionario de agresividad premeditada e impulsiva en adolescentes." Madrid: Tea Ediciones (2010).

[3] F. Scarpina and S. Tagini, "The stroop color and word test," Frontiers in psychology, vol. 8, p. 557, 2017.

[4] Heaton, Robert K., Gordon J. Chelune, Jack L. Talley, Gary G. Kay, and Glenn Curtiss. WCST: Wisconsin Card Sorting Test. Madrid, Spain:: TEA, 2001.

[5] J. A. Castro-Garc´ıa, A. J. Molina-Cantero, I. M. G´omez-Gonz´alez, S. Lafuente-Arroyo, and M. Merino-Monge, "Towards human stress and activity recognition: A review and a first approach based on low-cost wearables," Electronics, vol. 11, no. 1, p. 155, 2022.

[6] P. Sawangjai, S. Hompoonsup, P. Leelaarporn, S. Kongwudhikunakorn, and T. Wilaiprasitporn, "Consumer grade eeg measuring sensors as research tools: A review," IEEE Sensors Journal, vol. 20, no. 8, pp. 3996-4024, 2020.

Planned statistical analysis *(whenever possible, analyze data disaggregated by sex)*

The main objective of the work is to verify the effectiveness of the methodology.

proposal for the treatment of aggressive episodes. For this purpose, we will use the metrics derived from the tests used as inclusion criteria, which will be passed at the end of the intervention period, together with the variable indicating the number of weekly episodes. We will see if there are significant differences in these metrics before, after the intervention and with the control group. To this end, we will be able to study the process disaggregated by sex, in order to verify whether there are differences between genders, using the Kruskal-Wallis method, which does not require any prior hypothesis of verification of the homoscedasticity of the sample.

This same statistical analysis will be extended to the different characteristics that we will obtain from the physiological signals. In doing so, we seek to determine which of these characteristics or traits are influenced, in a significant way, by the aggressive episodes evoked in the clinic. The Kruskal-Wallis analysis will help us in this. We will also apply, in parallel, an exploratory factor analysis (EFA) in order to reduce the number of features and to be able to develop in the future a low-cost system that allows reliable monitoring of these events.

For the prediction of an aggressive act, and to help the subject to control aggressiveness in the future, we will proceed to analyze the 3 minutes prior to the outburst. A logistic regression will be applied to verify the detection capabilities of the system.

If it is a project in which some kind of **intervention** is carried out, explain what the intervention consists of, its development and the nature of the subjects' participation.

In the intervention, biofeedback (HR and EDA signals) will be applied, along with virtual reality and cognitive restructuring therapies [1] that have proven to be efficient for anger management. Such therapy is based on the fact that people minimize their negative feelings and behaviors when they are aware of how irrational they are and work to change their mind by continuously confronting them. The control group will receive the same cognitive restructuring therapy but without the support of virtual reality and biosignals.

Some sessions of the intervention group will be dedicated to the recognition of some physiological parameters before, during and after an aggressive behavior, and how these can be used, through biofeedback and virtual reality as a method to avoid an outburst. Psychological therapy will go in that direction, knowing how to address those feelings that lead to an episode of rage, before occurs, thanks to the recognition of the internal state through biofeedback. Specifically, what is proposed is that, through cognitive restructuring, we can modify the way we think and the way we think about the feelings that lead to an episode of anger.

interpret things. Many times, a good part of the problems are related to the impossibility of looking for alternative explanations of what is happening. Thus, cognitive restructuring can be interpreted as a strategy to improve patients' chances of modifying their cognitive schemas in the most adaptive way possible. In other , cognitive restructuring therapy is focused on the subject being able to identify the negative thoughts and beliefs presented in situations of conflict and/or frustration, which are the main triggers of explosive behaviors. They will be taught how certain physiological responses have been conditioned to certain cognitions that cause the person to lose self-control. Therefore, they are trained in the identification of the physiological response, the identification of the emotion that triggers and the negative thinking that accompanies them. The aim is that, through the identification of such thoughts and having a greater awareness of their emotions and physiological responses, the person will be dismantling these irrational thoughts.

EXAMPLE.

	ACONTECIMIENTO	EVALUACIÓN	EMOCIÓN
IRRACIONAL	“mi madre me dice que no puedo jugar a la play”	“sólo quiere fastidiarme la vida y que no tenga tiempo de disfrute”	Enfado, rabia, odio
RACIONAL	“mi madre me dice que no puedo jugar a la play”	“mañana tengo un examen que tengo que seguir trabajando”	Me molesta

Through cognitive restructuring therapy they are taught to evaluate situations more rationally. This coupled with biofeedback training, in which they learn to identify their physiological response, they understand that their emotional response is disproportionate.

[1] L. Canet-Juric, A. Garćıa-Coni, M. L. Andŕes, S. Vernucci, Y. Aydmune, F. Stelzer et al., "Intervention on cognitive, behavioral and emotional self-regulation in children: A review of process-based and school curriculum approaches, in Argentina," Revista Argentina de Ciencias del Comportamiento, vol. 12, no. 1, pp. 1-25, 2020.

If the project involves the **creation or validation of a scale or questionnaire**, indicate the procedure to be followed.

Not applicable

III.3. SCHEDULE *(estimated timing of the various phases of the project)*
Each session takes one week and lasts approximately 1 hour. Only the initial part of information gathering at home takes an undetermined amount of time, but in the range of 1 - 4 weeks. See the graph in the methodological development.

IV. DATA PROTECTION	
Data processing	*If the data will be anonymized or pseudo-anonymized. Procedure used*
The researcher Isabel Rojas, who, because of her profile, will be responsible for recruiting and carrying out the therapy, will know the names of the children/adolescents, but these names will not be given to the rest of the research staff, who will process the physiological data collected. Specifically, Isabel Rojas will create a label for each patient. The investigators will know the label but will have no further information about them. The label is necessary to be able to relate the different sessions to each patient. Therefore, the data are pseudo-anonymized at source for further processing.	
Custody, access y assignment from the data	*How the data will be stored and who will have access to the data*
The data will be stored in the University of Seville network for further study. Access to the data will only be allowed to the researchers of the project. The principal investigator will create a folder where all the records will be stored, and will give shared access to remaining investigators.	
Uses and purposes of the data	
To verify the efficacy of therapy, to analyze the effect of an aggressive flare on physiological signals and to determine the ability to predict a flare.	
Data retention period and data destruction procedure	
The data will be kept until the analysis of the data has been completed and a publication in a scientific journal has been made. The data will be destroyed one month after the publication of the results or when a family member explicitly requests their deletion. The videos will be deleted at the end of the experiment, when they are no longer needed.	
