# Supplementary material for: Evaluating the effectiveness of integrating biofeedback in the treatment of aggressive outbursts (BRET-IA2): A study protocol
Source: PLoS One. 2025 Jul 7;20(7):e0327361. doi: 10.1371/journal.pone.0327361 (PMC12233311; doi:10.1371/journal.pone.0327361)
Supplement: Attachment — Submitted filename: Response to Reviewers.pdf [file pone.0327361.s004.pdf]

Response to Reviewers and Editor's comments

First of all, the authors want to thank reviewers and editor for their useful comments, and for allowing the resubmission of this Study Protocol. Below is a copy of the issues detected by reviewers and editor that I will proceed to answer.

Answers to Editor's Comments

Editor #1.1: Please ensure that your manuscript meets PLOS ONE's style requirements, including those for file naming. The PLOS ONE style templates can be found at https://journals.plos.org/plosone/s/file?id=wjVg/PLOSOne_formatting_sample_main_body.pdf and https://journals.plos.org/plosone/s/file?id=ba62/PLOSOne_formatting_sample_title_authors_affiliations.pdf

Answer #1

We have already revised the format of the manuscript to meet the PLOS ONE's style.

Editor #1.2: We note that the grant information you provided in the 'Funding Information' and 'Financial Disclosure' sections do not match. When you resubmit, please ensure that you provide the correct grant numbers for the awards you received for your study in the 'Funding Information' section.

Answer #2

Done.

Editor #1.3: Thank you for stating the following financial disclosure:

"This research was funded by project Bret(IA)2, Grant PID2023-147508OB-I00 Funded by MCIN/AEI/ 10.13039/501100011033"

Please state what role the funders took in the study. If the funders had no role, please state: "The funders had no role in study design, data collection and analysis, decision to publish, or preparation of the manuscript." If this statement is not correct you must amend it as needed.P lease include this amended Role of Funder statement in your cover letter; we will change the online submission form on your behalf.

Answer #3:

Thank you for changing the online submission. We have included the information in the cover letter and added the role of funder statement.

Editor #1.4: Thank you for stating the following in the Acknowledgments Section of your manuscript: The authors would like to thank the participants' parents for their cooperation in this study and the INEBIR center for allowing us to use several rooms where we will deploy the materials needed for this research. This research was funded by project Bret(IA)2 , Grant PID2023-147508OB-I00 funded by MCIN/AEI/ 10.13039/501100011033, and by FEDER, UE.

We note that you have provided funding information that is not currently declared in your Funding Statement. However, funding information should not appear in the Acknowledgments section or other areas of your manuscript. We will only publish funding information present in the Funding Statement section of the online submission form.

Please remove any funding-related text from the manuscript and let us know how you would like to update your Funding Statement. Currently, your Funding Statement reads as follows:

This research was funded by project Bret(IA)2, Grant PID2023-147508OB-I00 472 Funded by MCIN/AEI/ 10.13039/501100011033

Please include your amended statements within your cover letter; we will change the online submission form on your behalf.

Answer #4

We have fixed the grant information and added the correct text to be uploaded to the online submission in the cover letter.

Editor #1.5: Your ethics statement should only appear in the Methods section of your manuscript. If your ethics statement is written in any section besides the Methods, please move it to the Methods section and delete it from any other section. Please ensure that your ethics statement is included in your manuscript, as the ethics statement entered into the online submission form will not be published alongside your manuscript.

Answer #5

We have moved the Ethics statement to the Methods section.

Editor #1.6: Please provide a complete Data Availability Statement in the submission form, ensuring you include all necessary access information or a reason for why you are unable to make your data freely accessible. If your research concerns only data provided within your submission, please write “All data are in the manuscript and/or supporting information files” as your Data Availability Statement.

Answer #6

The manuscript describes a study protocol so there are no data available at this moment.

Editor #1.7: Please amend your list of authors on the manuscript to ensure that each author is linked to an affiliation. Authors’ affiliations should reflect the institution where the work was done (if authors moved subsequently, you can also list the new affiliation stating “current affiliation:….” As necessary).

Answer #7

All authors are linked to their affiliations.

Editor #1.8: Please remove all personal information, ensure that the data shared are in accordance with participant consent, and re-upload a fully anonymized data set.

Note: spreadsheet columns with personal information must be removed and not hidden as all hidden columns will appear in the published file.

Additional guidance on preparing raw data for publication can be found in our Data Policy (https://journals.plos.org/plosone/s/data-availability#loc-human-research-participant-data-and-other-sensitive-data) and in the following article: http://www.bmj.com/content/340/bmj.c181.long.

Answer #8

There are no preliminary data in this study. We are considering creating a database after the experiment.

Editor #1.9: Please review your reference list to ensure that it is complete and correct. If you have cited papers that have been retracted, please include the rationale for doing so in the manuscript text, or remove these references and replace them with relevant current references. Any changes to the reference list should be mentioned in the rebuttal letter that accompanies your revised manuscript. If you need to cite a retracted article, indicate the article’s retracted status in the References list and also include a citation and full reference for the retraction notice

Answer #9

We have performed a thourogh revision of references to guarantee that it is complete and correct. Below are the new titles added according to reviewers' comments:

11. Chen WG, Schloesser D, Arensdorf AM, Simmons JM, Cui C, Valentino R, et al. The emerging science of interoception: sensing, integrating, interpreting, and regulating signals within the self. Trends in neurosciences. 2021;44(1):3–16.

12. D'Andrea W, Nieves N, Van Cleave T. To thine own self be true: interoceptive accuracy and interpersonal problems. Borderline personality disorder and emotion dysregulation. 2022;9(1):6. June 6, 2025 16/20

13. Feldman MJ, MacCormack JK, Bonar AS, Lindquist KA. Interoceptive ability moderates the effect of physiological reactivity on social judgment. Emotion. 2023;23(8):2231.

25. Karjalainen S, Kujala J, Parviainen T. Neural activity is modulated by spontaneous and volitionally controlled breathing. Biological Psychology. 2025;197:109026. doi:10.1016/j.biopsycho.2025.109026.

26. Baldini A, Patron E, Gentili C, Scilingo EP, Greco A. Novel VR-Based Biofeedback Systems: A Comparison Between Heart Rate Variability- and Electrodermal Activity-Driven Approaches. IEEE Transactions on Affective Computing. 2024;doi:10.1109/TAFFC.2024.3352424.

27. Jung H, Yoo HJ, Choi P, Nashiro K, Min J, Cho C, et al. Changes in Negative Emotions Across Five Weeks of HRV Biofeedback Intervention were Mediated by Changes in Resting Heart Rate Variability. Applied Psychophysiology and Biofeedback. 2025;50:25–48. doi:10.1007/s10484-024-09674-x.

31. Cohen J. The effect size. Statistical power analysis for the behavioral sciences. Abingdon: Routledge. 1988; p. 77–83.

53. Van Buuren S, Van Buuren S. Flexible imputation of missing data. vol. 10. CRC press Boca Raton, FL; 2012

56. Lane RD, Schwartz GE. Levels of emotional awareness: acognitive-developmental theory and its application to psychopathology. The American journal of psychiatry. 1987;144(2):133–143.

Answers to Reviewers' Comments

Reviewer #1.1: The introduction is well-developed; however, it lacks information on how biofeedback of peripheral signals (e.g., HRV, SCL) may influence central nervous system activity, as well as the role of interoceptive signal discrimination in the regulation of aggressive behaviors. Including this information could help provide a stronger rationale for the use of biofeedback as an intervention.

Answer #1.1

We would like to thank the reviewer for the suggestions. These have provided us with valuable information to enrich the introduction section. We have included some pieces of information in the introduction regarding interoception and the effect of bioffedback on peripheral signals.

Reviewer #1.2: The authors calculated the sample size based on a moderate effect size, yet they do not justify why this effect size was chosen. Please include a meta-analysis or previous study to support this choice. Moreover, they mention that the sample size calculation was based on a t-test, which compares the means between two groups. However, they later indicate that at least four groups will be formed, making the selected statistical test inappropriate. The sample size should be recalculated using the appropriate statistical test and providing a justification for the expected effect size. Additionally, consider the effect size for both between-group comparisons and within-subject comparisons.

Answer #1.2

We would like to clarify that the study includes two groups only: an intervention group and a control group. We set four subgroups during the recruitment to build balanced control and intervention groups according to gender and age. Therefore, the sample size calculation was appropriately based on a two-sample t-test, which compares means between two independent groups. We have now clarified this in the manuscript to avoid confusion.

Regarding the effect size, we selected a moderate effect size (Cohen's d = 0.5) as it represents a practically meaningful difference in the context of clinical decision-making, even if smaller effects may also be statistically detectable in larger samples and it is a commonly used convention in behavioral and clinical research when planning exploratory studies [Cohen, 1988]. For a small effect size (Cohen's d=0.2) we would need to recruit more than 300 participants, and we do not have human and material resources to complete this study in relativately short time.

We found a study that investigated whether pededing physiological and motion data measured by a reist-worn biosensor can predit aggression to others [Goodwin, 2019] and the study recruited only 20 young people.

[Cohen, 1988] Cohen J. The effect size. Statistical power analysis for the behavioral sciences. Abingdon: Routledge. 1988; p. 77–83.

[Goodwin, 2019] Goodwin, Matthew S., et al. "Predicting aggression to others in youth with autism using a wearable biosensor." *Autism research* 12.8 (2019): 1286-1296.

Reviewer #1.3: In the description of the sessions, it is unclear whether the evocation sessions will involve the same episode each time. Furthermore, in the biofeedback

session, it is not specified whether, beyond recognizing the interoceptive sensations associated with increased sympathetic activity, participants will also be taught regulatory skills using physiological feedback.

Answer #1.3:

We have cleared that the use of different videos to ellicit an outburst at laboratory. Regarding the therapy, the manuscript explains that during block 3 both control and intervention groups apply cognitive therapy. The difference between them is the use or not of biofeedback to enhance interoception.

Reviewer #1.4: The proposed statistical analyses (Mann-Whitney and Wilcoxon) are not adequate for comparing the four proposed groups. These tests also do not allow for testing interactions between factors. It is recommended to propose a parametric analysis and only mention the possibility of non-parametric tests if data do not meet the necessary assumptions for parametric analysis.

Answer #1.4

We would like to clarify that the study does not involve four groups, but rather two conditions compared within the same participants (i.e., a repeated measures design). Therefore, the Wilcoxon signed-rank test was proposed as the primary statistical method to compare paired samples when the assumptions for parametric tests are not met. The Mann-Whitney U test was mentioned in the context of potential between-group comparisons in exploratory analyses, but it is not central to the primary analysis.

Nonetheless, in accordance with the reviewer's suggestion, we have revised the manuscript to indicate that a parametric test will be used if the assumptions of normality and homogeneity are satisfied. The non-parametric alternative (Wilcoxon) is now presented as a contingency plan should those assumptions not hold.

We thank the reviewer for highlighting the importance of clearly specifying the statistical approach.

Reviewer #1.5: The authors refer to Freud's psychoanalytic theory in the discussion section. This theory not only lacks scientific support but is also unrelated to the authors' proposed intervention, which follows a cognitive approach.

Answer #1.5

Thank you for your insightful observation. We acknowledge that the mention of Freud's psychoanalytic theory in the discussion section may not be directly relevant to the cognitive framework underpinning our intervention.

In light of this, we have removed the reference to psychoanalytic theory to maintain conceptual and methodological consistency with the cognitive approach adopted in our study. We appreciate your feedback in helping us ensure a clearer and more focused discussion.

Reviewer #1.6: The role of various brain structures in the regulation of aggressive behaviors is discussed, but no hypothesis is offered regarding the potential mechanism of change when training interoceptive awareness through biofeedback. Please include such a hypothesis in the discussion section.

Answer #1.6

Thank you for this important observation. We agree that providing a plausible hypothesis regarding the mechanism of change would strengthen the discussion. We have now added a theoretical explanation suggesting that interoceptive awareness training via biofeedback may modulate activity in brain regions involved in emotion regulation—particularly the **insula**, **anterior cingulate cortex**, and **prefrontal areas**—which in turn can reduce dysregulated aggressive responses. These structures are known to support both interoception and top-down control over emotional and behavioral impulses. The revised discussion includes this hypothesis with appropriate references.

Reviewer #1.7: If this is acknowledged in advance, why not include more biofeedback sessions? In fact, it would be easy to incorporate them into all sessions for the experimental group. Consider modifying the protocol to include additional biofeedback sessions.

Answer #1.7

We acknowledge the concern regarding the duration of biofeedback exposure and its potential impact on therapeutic outcomes. The decision to limit the number of biofeedback sessions was based on several considerations, including time constraints, feasibility in real-world clinical settings, and the need to maintain participant engagement throughout the intervention.

While we recognize that more sessions might enhance familiarity with physiological signals, our protocol integrate biofeedback into key moments of the intervention to

optimize learning and transferability. Furthermore, the sessions are supported with self-monitoring tools to reinforce interoceptive awareness beyond the formal biofeedback training.

If the results indicate insufficient exposure, we will consider protocol adjustments in future trials. For the current study, however, we believe the structure keeps a balance between scientific rigor and practical applicability.

Reviewer #2.1: The sample size section is too scanty and lacks core details. What effect size was used to arrive at the final sample size of 62? What sample size estimation technique was used? Did the authors account for the non-response rate? Details must be provided

Answer #2.1

As explained now in the manuscript, the sample size was calculated based on a two tailed t-test for independent means, assuming a moderate effect size (Cohen's $d = 0.5$), $\alpha = 0.05$, and power = 0.80. This resulted in a required sample of 62 participants (31 per group). To account for potential non-response or dropout, we included an additional 10%, bringing the final target sample size to 70 participants. This approach ensures sufficient power to detect differences between groups while anticipating common challenges in recruitment and retention.

It is important to account for the non-response rate and we did not do it in the document. Thank you for this important observation.

Reviewer #2.2: It is a bit problematic to remove an outlier based on a threshold. It is also possible that there may be genuine values that are outliers based on the threshold defined by the interquartile range specified. If you just remove these outliers, your results may be biased. Once you identify an outlier based on the interquartile range threshold, you need to investigate the source of the outlier before you can determine the appropriate solution.

Answer #2.2

We agree that automatically removing outliers based solely on an interquartile range (IQR) threshold can be problematic, as it risks excluding valid data that may reflect true variability. In response to this observation, we have revised our approach to outlier handling. Instead of removing them directly, we carefully examined each identified outlier to assess whether it could be attributed to measurement errors, technical issues,

or specific characteristics of the participant. Data points were excluded only when a clear justification existed (e.g., confirmed recording errors).

We have updated the manuscript to reflect this more cautious and transparent approach to outlier treatment.

Reviewer #2.3: Under statistical analysis, the authors have already assumed that the residual of the outcome measure (assessment of cognitive therapy with biofeedback) will not be normally distributed. Therefore, they proposed the Mann-Whitney-Wilcoxon test, a non-parametric method, instead of the one-way ANOVA (a parametric test). Since this is just a protocol, parametric and non-parametric alternatives should be proposed. The same applies to Friedmann's test. For study protocols, I humbly believe that presenting parametric and non-parametric approaches is the way to go, as authors can only determine the true distribution of the outcome measures when they have data.

Answer #2.3

Thank you for your insightful comment. We agree that assuming normality in outcome measures may not always be appropriate, especially prior to data collection.

To address this, we have updated the manuscript to clarify that for outcomes not normally distributed, we will report the **median** and **interquartile range (IQR)** as summary measures. Normality will be assessed using graphical methods (e.g., histograms, Q–Q plots) and formal statistical tests (e.g., Shapiro–Wilk test) before deciding on the appropriate descriptive and inferential statistics.

These additional descriptions have been included in the revised version of the protocol.

Reviewer #2.4: There should be a section that summarizes the primary and secondary outcome measures and the measurement scale of these outcome measures (continuous, binary, count, ordinal, nominal etc).

Answer #2.4

This section has been added to the manuscript. However, as there is a total of 40 psychometric values, this section only contains a summary of the primary measures. If necessary, we could include all the secondary variables in an appendix at the end of the manuscript.

Reviewer #2.5: How would the authors address issues of missing values or observations after the implementation of the intervention? This must be included in the protocol

Answer #2.5:

We agree that this point is important. We have added a subsection at the end of the Analysis section when we describe the most important strategies to handle with missingness. Basically, if there is more than 5% of missing data, we will apply a multiple imputation method to generate several complete datasets by predicting missing values based on observed variables. A post sensibility analysis will also be applied to compare the outcomes under different conditions and assess the robustness of the findings.
