# Supplementary material for: Evaluating the effectiveness of integrating biofeedback in the treatment of aggressive outbursts (BRET-IA2): A study protocol
Source: PLoS One. 2025 Jul 7;20(7):e0327361. doi: 10.1371/journal.pone.0327361 (PMC12233311; doi:10.1371/journal.pone.0327361)
Supplement: Attachment — Submitted filename: Answers to Reviewers1.pdf [file pone.0327361.s005.pdf]

Answers to Reviewers' Comments

First of all, the authors want to thank reviewers for their comments and the Editor for allowing the resubmission of this Study Protocol. Below is a copy of the issues detected by reviewers that I will proceed to answer.

Reviewer #1: As described here, the paper does not talk about the biofeedback in an integrated manner. It doesn't properly explain or describe the context of the VR treatment. It doesn't read like a Study Protocol, rather it reads more like sections of a thesis that were pulled together in to a 'Study Design' paper. The paper is disjointed in places, and many sections are missing proper explanations, remember it needs to read like a paper, not just a presentation of facts. The paper requires a rewrite with a stronger focus on the Study Protocol. I recommend the authors consult:

https://trialsjournal.biomedcentral.com/submission-guidelines/preparing-your-manuscript/study-protocol
https://pmc.ncbi.nlm.nih.gov/articles/PMC6172884/
https://www.who.int/groups/research-ethics-review-committee/recommended-format-for-a-research-protocol

Answer #1.1

We would like to thank the reviewers for their suggested readings. These have provided us with valuable insights into this type of publication and the varying requirements of different editors.

The original paper described a study protocol aimed at assessing the effect of biofeedback on managing aggressive outbursts, along with several secondary objectives. However, the detailed description of the methods required to achieve these secondary objectives added complexity to the paper and, at times, made it resemble a Study Design. For these reasons, we decided to remove all secondary objectives and focus solely on the main aim.

Regarding the concept of biofeedback, it is clearly defined on the first page, in the abstract. Virtual Reality is used only to display physiological data to participants or during specific relaxation phases to help them shift focus and calm down.

Specifics:

Line 29: GRAMMAR: (amygdala, dorso-lateral PFC, hypothalamus, ACC, ...), should

be rewritten to say (for example, the amygdala, dorso-lateral PFC, hypothalamus, etc.).

Answer #1.2 We have performed the suggested changes.

Line 47 and in other areas: name the authors when referring directly to their work, don't just list a citation number.

Answer #1.3 The whole manuscript has been thoroughly reviewed to avoid citing the reference number, as suggested. All direct citations have been modified.

Line 56: CLARITY: Be precise, a later onset

Answer #1.4 The sentences have been slightly changed for the sake of clarity. "The onset of aggressive behavior at earlier ages is linked to higher biological risks and often results in life-course persistent antisocial behavior. A better long-term prognosis, leading to a relatively normal daily life, is more likely when aggressive behavior emerges at older ages (17).".

Line 74: CLARITY: "they" - Who?

Answer #1.5
After restructuring the section, the paragragh containing this sentence was removed.

Lines 88 - 102. COMMENT: This reads like a hypothesis and research questions from a thesis, not properly framed or presented for a paper.

Answer #1.6
We have eliminated the hypothesis list from the manuscript.

Line 249: CLARIFICATION: Actually define (spell out) BR and ST in the following paragraph, don't just jump in to technical description of sensors.

Answer #1.7 Some important changes include the introduction of new equipment and the removal of the section containing the acronym. Nevertheless, we have reviewed the entire document to ensure the acronym is introduced upon its first use. To achieve this, we utilized the LaTeX acronym package, which automates this process.

Line 282. COMMENT: Oculus Rift CV1 is a very old and outdated hmd.

Answer #1.8 Reviewer #1 is right. Indeed, when additional units were purchased for the experiment, we discovered that the model was obsolete. We have replaced it with the Meta Quest 3.

Table 1. HR-variables. CLARIFICATION: Define NN intervals, SDNN, pNN50 - don't assume your reader understands technical acronyms.

Answer #1.9 Since the manuscript has been restructured, this information no longer appears in the new version.

Line 343. CLARIFICATION: Why 2 second window? Explain.

Answer #1.10 Since the manuscript has been restructured, this information no longer appears in the new version.

Table 2. EDA-variables. CLARIFICATION: Explain SCR, ISCR and Tonic for the reader.

Answer #1.11 This table has been removed from the document because it shifted the focus of the main aim. We will only use SCL for biofeedback, which has been explained.

Line 349: TYPESETTING: Equation number is missing

Answer #1.11 Since the manuscript has been restructured, this information no longer appears in the new version.

Line 430: CLARIFICATION: This is the first time you've mentioned AI. Explain the basis for choosing the particular ML algorithms.

Answer #1.12 Since the manuscript has been restructured, this information no longer appears in the new version.

Missing a few key sources in the biosensors in VR therapy field.

Answer #1.13 Virtual Reality is used to display HR, HRV, and SCL to the participant and to facilitate relaxation. References (27–29) have been added to support these aspects.

"After the basal register phase, teenagers move on a relaxation
phase in which they use an immersive Virtual reality (VR) system for 10
minutes. In this phase, participants can move into a landscape while
listening to ambient sounds (birds and sea waves). VR, and particularly
immersive virtual reality (IVR), have demonstrated their effectiveness in

therapy with people with behavioral disorders, improving the recognition of emotions, social skills, and attention, while diminishing their anxiety and phobias (27; 28; 29).”

Reviewer #2: Important note: This review pertains only to ‘statistical aspects’ of the study and so ‘clinical aspects’ [like medical importance, relevance of the study, ‘clinical significance and implication(s)’ of the whole study, etc.] are to be evaluated [should be assessed] separately/independently. Further please note that any ‘statistical review’ is generally done under the assumption that study specific methodological [as well as execution] issues are perfectly taken care of by the investigator(s). This review is not an exception to that and so does not cover clinical aspects {however, seldom comments are made only if those issues are intimately / scientifically related & intermingle with ‘statistical aspects’ of the study}. Agreed that ‘statistical methods’ are used as just tools here, however, they are vital part of methodology [and so should be given due importance]. I look at the manuscript in/with statistical view point, other reviewer(s) look(s) at it with different angle so that in totality the review is very comprehensive. However, there should be efforts from authors side to improve (may be by taking clues from reviewer’s comments). Therefore, please do not limit the revision only (with respect) to comments made here.

COMMENTS: I have different opinion / observations/concerns or rather questions regarding quite a few issues [including some serious ones] which are given below:

First of all, I regret to point out the manuscript file is in (.pdf) format. Manuscript file in [MS-WORD i.e., in (.doc/x)] format is convenient to/for reviewer(s) to reproduce [copy-paste] some material to highlight/pin-point required/desired changes/modifications and for word count, etc.

Secondly, I noted that your ABSTRACT is drafted alright (in my opinion), but is ‘assay type’. It is preferable to divide the ABSTRACT with small sections like ‘Objective(s)’, ‘Methods’, ‘Results’, ‘Conclusions’, etc. which is an accepted practice of most of the good/standard journals [including this one, though ‘The PLoS One Guidelines to Authors’ did not specify an Abstract format, it is desirable]. It will definitely be more informative then, I guess, whatever the article type may be {though Section headings may differ for different Article Types [example: Study Protocol]}.

{Note that your ‘ABSTRACT’ is little shorter (192 words) than desired and there is nothing on/about ‘METHODOLOGY’.

Answer #2.1 The abstract has been structured and its length increased according to rhw suggestions.

Moreover, considering ADHD and 'antisocial disorder' together (in one bracket) may not be correct. In my opinion, 'impulsive behavior' and quality/type of 'aggressiveness' are different in these two (ADHD and 'antisocial disorder'). Are not they? Agreed that both are with impulsive behavior, I guess their impulsivity is of different kind. [May please confirm from expert psychiatrist].}

Answer #2.2 Indeed, that paragraph was confusing and has been removed. That is why we focus on aggressive (and impulsive) behavior, regardless of the diagnosis.

Next, account given on sample size estimation in lines 112-4 [The sample size has been estimated based on the fact that about 6% of the population has shown impulsive/aggressive behaviors related to lack of anger control [24, 25] and the use of the Cochrane formula, when considering a confidence level of 10% and a precision of 5%] is of doubtful value because in lines 78-79 you stated that 'This study aims at assessing the use of biofeedback on the treatment of aggressive impulse in the young population with ADHD or antisocial disorder' and we consider a confidence level & a precision only in 'estimation' study (not in hypothesis testing study).

Answer 2.3 The reviewer is absolutely right. We have changed the estimation of the sample size according to hypothesis testing for a confidence level of 0.05, a test power of 0.8 and a moderate effect size.

Moreover, reason mentioned [lines 107- 110: the early onset of aggressive behavior is linked to higher biological risks and often leads to persistent antisocial behavior, highlighting the urgency of applying treatment as soon as possible. For this reason, the group to be studied will consist of 60 participants aged 10-16 years] is wonderful {questionable}, and surprisingly completely irrelevant (in fact, it is not very clear whether the study limits only to 'estimation' or you are also interested in hypothesis testing).

Answer 2.4 Maybe the explanation lacks from clarity. There is a study that poses that the earlier the aggressive behavior appears the worse the long-term effects. Therefor, its very important to act as soon as possible. The redaction of that text segment has been changed.

In lines 88-89 you stated that "The main hypothesis of this study is that there is a preparatory stage in the body that anticipates the imminent onset of an aggressive outburst." Is this the

main hypothesis or the main assumption?

Answer 2.5: None of them. It is possible to predict the onset of an aggressive outbursts one minute before its onset. Predicting aggression to others in youth with autism using a wearable biosensor - PubMed

In lines 148-149 it is stated that "Participants will be randomly divided into 2 groups: control (children who will not receive the experimental treatment based on biofeedback) and the intervention group." Why the 'design' of the study is mention so late in the manuscript? In my opinion, "Design of the study" is of vital importance and be made clear in the 'ABSTRACT'. Further, you stated that "The same number of subjects will be included in each group" but not discuss the method of 'randomization'. You may know that 'Permuted Block Randomization' ensures same group sizes (not simple randomization) and that randomization is a process [not only sequence generation] which includes 'Allocation Concealment'.

Answer 2.6 The type of experiment to conduct has been now detailed in the abstract. Additionally, to reduce bias in the control/intervention groups, we have stratified by age and gender.

Kindly check for the 'English' language [defects are seen troughout]. Agreed that English is not our mother tongue (definitely not mine, may or may not be yours but certainly not of many readers), however in any case, remember/ Kindly mind you [please excuse me for such a harsh comment/statement] that this is a scientific/academic document and so all details should be clearly/correctly communicated (do not take reader's for granted). You may take help of language professional expert, if needed.

Answer #2.7 The manuscript has been reviewed by the translation services of the University.

In 'Analysis' section [lines 404-444] many techniques/methods/statistical tests are mentioned/listed; however, the entire section is full of confusion. Is not the statement made in lines 232-3 [that "The differences between the new scores, with respect to the initial ones, along with the differences between control and intervention groups, will be used to assess the efficacy of the intervention (RQ1)"] confusing? In my knowledge, to compare two groups use of Mann-Whitney test (non-parametric) is desired whereas the Kruskal-Wallis test is indicated if three or more groups are involved. But you stated in lines 409-411 that "These differences will be compared between interventions and control to ascertain statistical significance. To do that, we use the Kruskal-Wallis test, a non-parametric method for testing whether samples originate from the same distribution". Is that correct? In fact, these testing should be done on 'CHANGE SCORE' which is not mentioned anywhere. In lines 414-415 you stated "The Friedman test will verify the statistical significance of the EDA and ECG features between the two

scenarios: home and clinic (RQ4)" which is not understood. For 'comparison' between two one-to-one matched or paired groups, we generally use Wilcoxon's matched-paired rank test (non-parametric). Friedman test is indicated only when three or more (one-to-one matched or paired) groups are involved.

Answer #2.8 We have modified the analysis according to the suggestions. Although the Kruskal-Wallis test works well with two groups, it is more appropriate to use Wilcoxon's test. The Friedman test has been maintained because there are repeated measures across several sessions.

Moreover, limitations (if any) of the study are not mentioned/listed anywhere. Does that mean {according to authors} there are none?

Answer #2.9 The following limitations have been added to the manuscript:

The application period of biofeedback therapy, along with the time required to recognize the physiological variables used to identify the preparatory state that precedes an aggressive outburst, might be too short to achieve significant results to validate the therapy.

If an aggressive impulse involves numerous movements by the individual, it is possible that the capture of physiological signals could be significantly disrupted, and consequently, the extraction of features derived from those signals, which in turn may affect the detection of the preparatory state.

There may also be an environmental inhibition effect when attempting to provoke an impulsive response in the laboratory. This could be a serious limitation, as it might significantly reduce the amount of valid data available for training artificial intelligence models, thereby diminishing the power of the proposed statistical analysis.

The equipment used to measure physiological signals could be damaged during aggressive responses. Although replacement materials have been planned, they might be insufficient, requiring additional budget and time to conduct the tests.

As pointed out in 'important note' above "This review pertains only to 'statistical aspects' of the study and so 'clinical aspects' should be assessed separately/independently [one should carefully consider/look at the clinical implications of the study]. In my opinion, to rescue this article (which seems quite difficult, if not impossible), large amount of re-vision (re-drafting)

may be needed. However, please do not limit the revision only (with respect) to comments made here. More improvement is expected. How to handle/accommodate these suggestions is not questionable as the study is in protocol stage. ‘Major revision’ is recommended [in lieu of plain rejection, to give chance to authors for improvement of the manuscript (because the study has potential) if they are ready/willing].
